# Comparative genome analysis of *Wolbachia* strain *w*Au

**DOI:** 10.1186/1471-2164-15-928

**Published:** 2014-10-24

**Authors:** Elizabeth R Sutton, Simon R Harris, Julian Parkhill, Steven P Sinkins

**Affiliations:** Department of Zoology and Peter Medawar Building for Pathogen Research, Nuffield Department of Medicine, University of Oxford, Oxford, UK; Pathogen Genomics, Wellcome Trust Sanger Institute, Wellcome Trust Genome Campus, Hinxton, Cambridge UK; Biomedical and Life Sciences, Lancaster University, Lancaster, UK

**Keywords:** *Wolbachia*, *w*Au, *w*Mel, Genome, Cytoplasmic incompatibility, Prophage, Transcriptional regulator, PacBio sequencing

## Abstract

**Background:**

*Wolbachia* intracellular bacteria can manipulate the reproduction of their arthropod hosts, including inducing sterility between populations known as cytoplasmic incompatibility (CI). Certain strains have been identified that are unable to induce or rescue CI, including *w*Au from *Drosophila*. Genome sequencing and comparison with CI-inducing related strain *w*Mel was undertaken in order to better understand the molecular basis of the phenotype.

**Results:**

Although the genomes were broadly similar, several rearrangements were identified, particularly in the prophage regions. Many orthologous genes contained single nucleotide polymorphisms (SNPs) between the two strains, but a subset containing major differences that would likely cause inactivation in *w*Au were identified, including the absence of the *w*Mel ortholog of a gene recently identified as a CI candidate in a proteomic study. The comparative analyses also focused on a family of transcriptional regulator genes implicated in CI in previous work, and revealed numerous differences between the strains, including those that would have major effects on predicted function.

**Conclusions:**

The study provides support for existing candidates and novel genes that may be involved in CI, and provides a basis for further functional studies to examine the molecular basis of the phenotype.

## Background

*Wolbachia pipientis* is a maternally inherited intracellular bacterium that infects a very large number of arthropod and nematode species [1] and can induce a variety of reproductive manipulations in arthropods to confer a selective advantage on infected females, thus promoting population invasion [2]. The most common manipulation is known as cytoplasmic incompatibility (CI) [2]. In the simplest form of unidirectional CI, uninfected females produce inviable offspring when mated with *Wolbachia*-infected males, while *Wolbachia*-infected females produce viable offspring when mated with both infected and uninfected males. Bidirectional CI can also occur, in which *Wolbachia*-infected females are incompatible with males infected with a different *Wolbachia* strain. At present little is known about the molecular mechanisms of CI, and this represents a critical roadblock in our understanding of *Wolbachia* biology. Identification of CI genes would also be beneficial for disease control applications using *Wolbachia*. Some *Wolbachia* strains have been found to block or reduce transmission of human viruses [3–7] and parasites such as filarial nematodes and *Plasmodium* [3, 8–11]; use of inhibitory *Wolbachia* for population replacement has shown considerable promise in field trials [12].

One approach to identifying genes involved in CI is genomic analysis of *Wolbachia*, comparing closely related incompatible strains with each other, or comparing CI-inducing strains with related strains that do not induce CI. In *D. simulans* the *Wolbachia* variant *w*Au expresses neither sperm modification in males, nor rescue of CI in females [13–17]; in other words *w*Au is ‘mod- resc-’, while CI-inducing strains are designated mod + resc + [2]. The *w*Au strain has, however, been shown to provide its *Drosophila* host with a degree of protection against the effects of pathogenic viruses [18]. The *w*Mel strain, from *D. melanogaster*, is most closely related to *w*Au and does induce CI; thus genes differing between these strains are candidates for involvement in CI [19]. The genome sequence of *w*Mel has already been reported [20], so here we undertook *w*Au genome sequencing in order to enable a comparative analysis.

Although the genomes of several *Wolbachia* strains have been published [20–26], acquisition of sequence data has been limited by the difficulty in obtaining a sufficient quantity and purity of *Wolbachia* genomic DNA (gDNA). *Wolbachia* are obligate endosymbionts that cannot be cultured outside of their hosts, and are often present in relatively low abundance. Obtaining enough gDNA has thus required time-consuming amplification and purification protocols to minimize contamination with host gDNA. In addition, assembly has been complicated by numerous repeated sequences. Here we utilised the Pacific Biosciences (PacBio) RS II platform for sequencing; the long reads generated by this technology facilitate assembly through genomic repeats.

Previous comparative analysis of the genomes of mutual incompatibility-generating *Wolbachia w*Pip sub-strains infecting *Culex pipiens* mosquitoes [21, 22] revealed highly similar genomes with a small number of whole gene differences. Most notably this included a transcriptional regulator gene designated *wtrM* identified in *w*Pip from *Cx. molestus* (*w*PipMol) but absent in *w*Pip from *Cx. quinquefasciatus* Pel and JHB (*w*PipPel and *w*PipJHB), which are bidirectionally incompatible with *Cx. molestus* [22]. Transfection of *Cx. quinquefasciatus* females with *wtrM* resulted in significant upregulation of *CPIJ005623*, a host gene implicated in CI based on knockdown studies [22]. Eight paralogous putative transcriptional regulator genes are present in *w*Mel (*WD0254*, *WD0255*, *WD0296*, *WD0508*, *WD0622*, *WD0623* and *WD0626* and *WD0627*). A specific comparison of these transcriptional regulator genes in *w*Mel and their homologs in *w*Au was therefore conducted to further investigate the hypothesis that disruptions to these genes could be responsible for the different CI phenotypes of these strains.

## Results and discussion

### Genomic DNA purity assessment

Approximate calculations based on quantitative PCR (qPCR) C(t) values for *w*Au and host genes were performed to estimate the degree of contamination with host gDNA in *w*Au gDNA samples extracted from cultured cells and whole adult flies. The estimated purity of *w*Au gDNA was ~60% for the extract from cultured cells, and >90% for the extract from whole adult flies. The latter is comparable to the figure of up to 97% reported previously [27] using the same extraction method. There is no previous data on *Wolbachia* gDNA extraction from cultured cells. One explanation for the lower purity could be that *Wolbachia* densities may be lower within cultured cells than *in vivo*.

### Genome sequencing and assembly

*w*Au genome sequencing was initially performed using the Illumina platform on gDNA extracted from whole adult files. However, the resulting assembly was fragmented in the regions of most interest, with scaffold positions uncertain. A second round of sequencing was therefore performed using the PacBio RS II system to obtain longer reads in an attempt to improve the assembly, using gDNA extracted from cultured cells rather whole adult files. The Illumina data was used to correct errors in the PacBio reads, which assembled into a single contig.

The achievement of a single contig assembly shows that PacBio represents an extremely useful new sequencing platform for rapid generation of finished bacterial genome assemblies. Furthermore, the generation of this single contig from a very small amount of DNA (approximately 2 ng), containing a substantial amount of host DNA contamination (~40%), suggests that PacBio is well suited to use in cases where it is hard to obtain a large amount of gDNA, including obligate endosymbionts, like *Wolbachia*, that cannot be cultured outside of host cells. The sequence generated was largely consistent with data produced using the Illumina platform, with only one single nucleotide polymorphism (SNP) between the two datasets. There were 88 indels relative to Illumina data; these were mostly single nucleotide insertions in the PacBio sequence, and were located in homopolymeric tracts, regions that are known to be prone to insertion errors in PacBio sequencing [28, 29]. These were corrected after mapping the Illumina reads to the PacBio assembly. Combining the PacBio reads with the shorter but more accurate Illumina reads was found to be a very useful approach, consistent with other findings [30, 31].

The use of cultured insect cell lines to obtain gDNA for genome sequencing represents a methodological departure from previous studies. All previous *Wolbachia* genomes have been sequenced using gDNA extracted directly from their native hosts. It is a time-consuming and often laborious process to rear sufficient numbers of the host insects for *Wolbachia* gDNA extraction, particularly for species with demanding rearing requirements. Transinfection of cells with *Wolbachia* is fairly easy to achieve, and amplification of cells to a suitable number is easier, quicker, and requires less space than whole organisms; this study used 24 flasks of cells, which were generated from a single flask in a few weeks. Concerns that the sequence of the *w*Au from cultured cells might have accumulated differences compared to the *w*Au genome found in flies, due to a relaxation in cell lines of the selective pressures that apply in its native host, were alleviated by the observation of only one SNP between the sequence obtained using *w*Au from cultured cells and that using *w*Au from its native host. It is possible that after a longer period of time more differences from *Wolbachia in vivo* would accumulate, so use of recently generated *Wolbachia*-infected cell lines, as employed here, is advisable.

### *w*Au genome features

**Figure 1:**
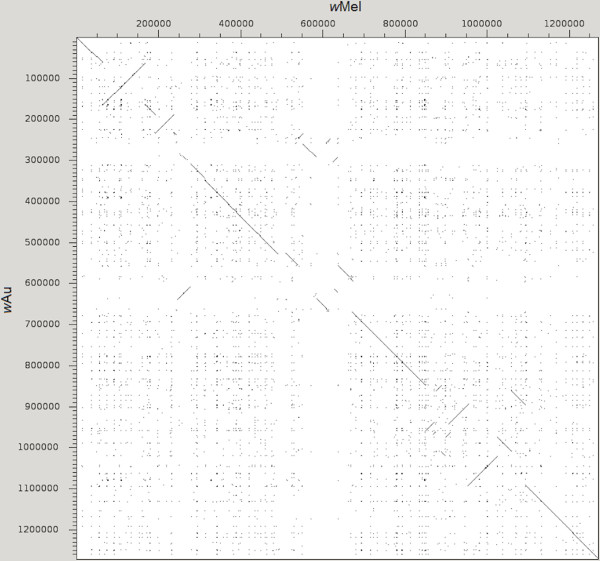
**Similarity between** ***w*** **Au and** ***w*** **Mel genomes.** A dot plot illustrating the similarity between *w*Au and *w*Mel genomes. Axes show position in the genome (bp). Lines indicate regions of similarity. Lines on the diagonal from top left to bottom right indicate regions with the same location and arrangement in both genomes. Lines in the same orientation as this diagonal but located elsewhere indicate regions that are translocated in one genome relative to the other. Lines at right angles to the diagonal indicate regions that are inverted in one genome relative to the other. Parallel lines indicate repeated or similar regions.

The *w*Au genome is a single circular chromosome of 1,268,461 bp. It has 1266 predicted genes, corresponding to a coding content of 84%. The major features of the genome, along with those of the *w*Mel genome, are shown in Table 1. Overall, the *w*Au and *w*Mel genomes are similar, but with a significant amount of rearrangement (Figure 1).

**Table 1:** **General features of** ***w*** **Au and** ***w*** **Mel genomes**

	***w***Au	***w***Mel
**Genome size (bp)**	1,268,461	1,267,782
**G + C content (%)**	35.22	35.23
**Predicted CDSs**	1266	1195
**Coding density (%)**	83.9	80.2
**Average gene size (bp)**	840	850
**Transfer RNAs**	34	34
**Ribosomal RNAs**	1 of each	1 of each
**Prophage regions**	3	3

Like *w*Mel, there is a large amount of DNA corresponding to mobile genetic elements in the *w*Au genome, including numerous insertion sequence (IS) elements. For example, 27 putative IS5 elements were identified, although most are likely to be inactive due to mutations or frameshifts. Some elements appear to have been active since the divergence of *w*Mel and *w*Au, as their locations in the genome differ between the two strains. In some cases their movement has resulted in disruption of genes in one strain. In many cases where there are structural differences between the genomes of the strains, it seems that mobile elements have provided a mechanism for the rearrangement.

### Comparison of prophage regions

**Figure 2:**
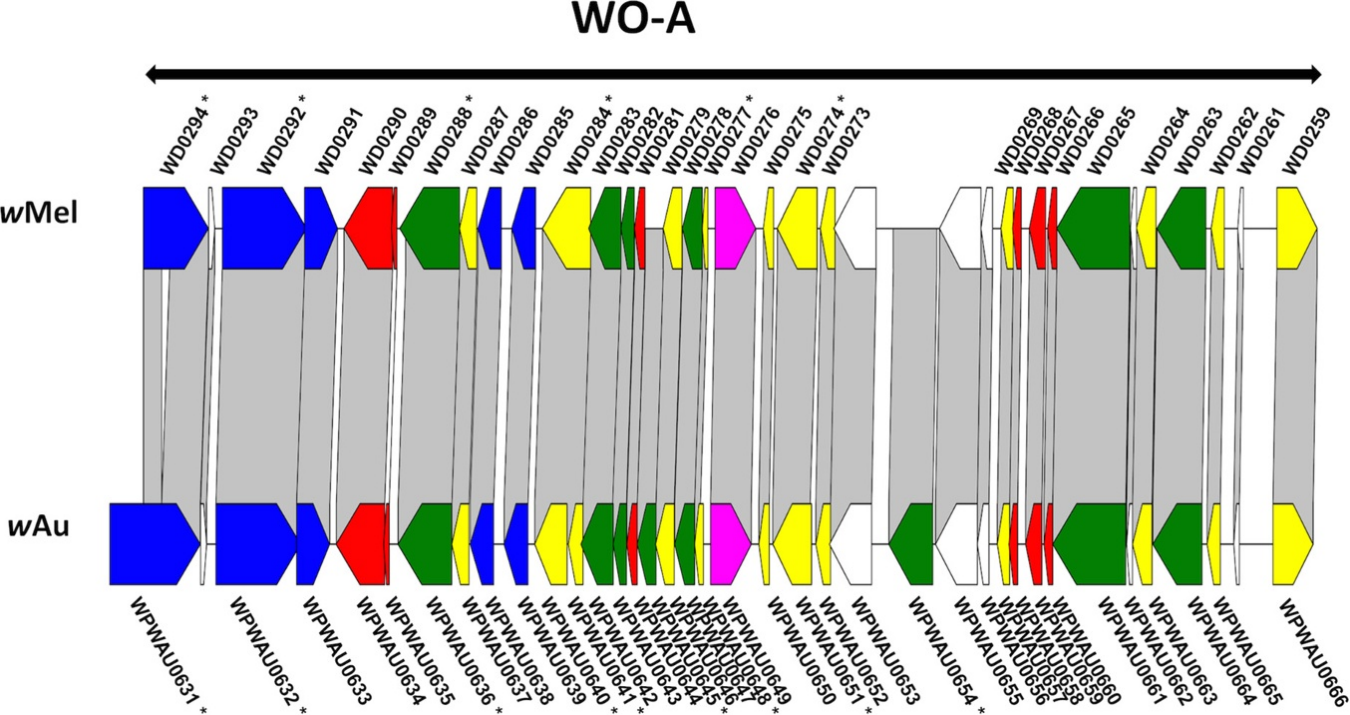
**Comparison of WO-A in** ***w*** **Au and** ***w*** **Mel.** Alignment of the WO-A prophage region between wAu and wMel. Matching sequences corresponding to predicted CDSs, identified using ACT and Geneious alignments, are connected by grey blocks. Genes whose sequences differ between strains such that a CDS is not predicted in one strain are not represented in the strain lacking the predicted CDS, but their corresponding sequences are still connected to the CDSs in the other strain by grey blocks. The double-headed black arrow indicate regions that have been drawn inverted relative to their orientation in the genome, for clarity of alignment visualisation. Asterisks indicate genes with differences other than SNPs between *w*Au and *w*Mel. Internal indels less than 20 bp in size are not shown. Predicted CDSs are colour coded as follows: green, phage structural or replication genes; yellow, conserved hypotheticals; red, hypotheticals; blue, ankyrin repeat genes; magenta, transposases or reverse transcriptases. White arrows indicate sequences that are not annotated in one of the two strains and are probable pseudogenes or mis-annotations. Predicted CDSs that result from interruptions, frameshifts or nonsense mutations, which are combined into a single CDS in the other strain, are coloured the same as the CDS from which they are derived, even though they may also be pseudogenes.

**Figure 3:**
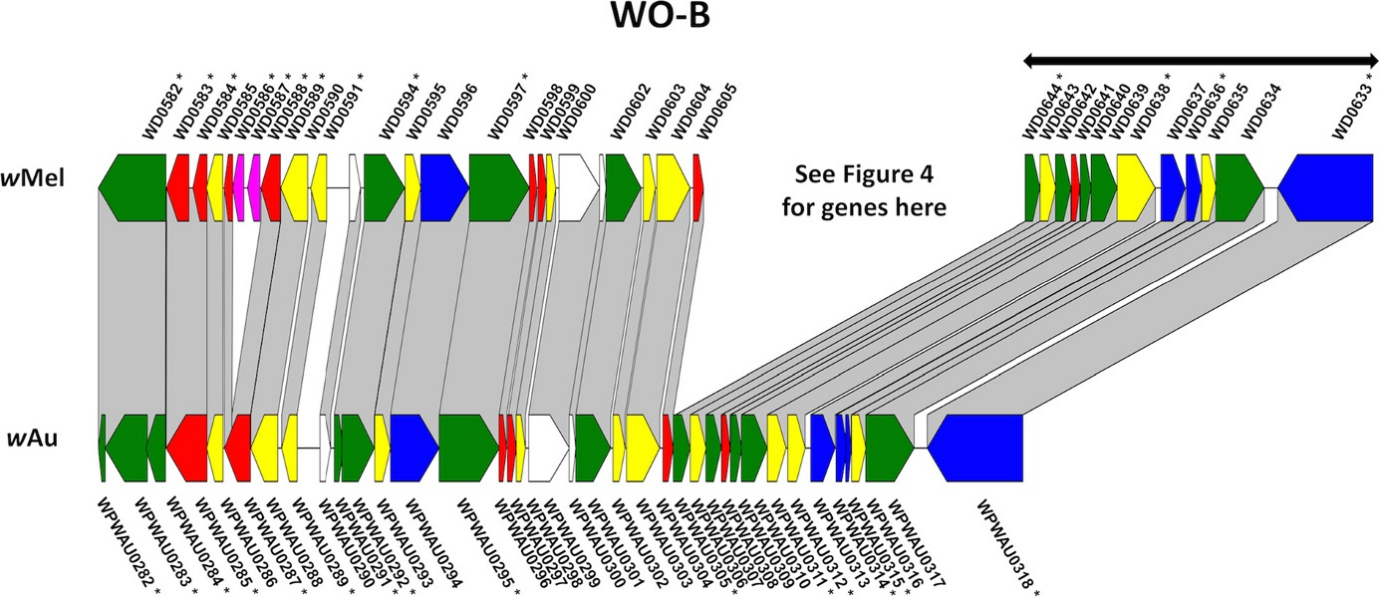
**Comparison of WO-B in** ***w*** **Au and** ***w*** **Mel.** Alignment of the WO-B prophage region between *w*Au and *w*Mel, constructed and presented as for Figure 2, with the same CDS colour coding, namely: green, phage structural or replication genes; yellow, conserved hypotheticals; red, hypotheticals; blue, ankyrin repeat genes; magenta, transposases or reverse transcriptases.

**Figure 4:**
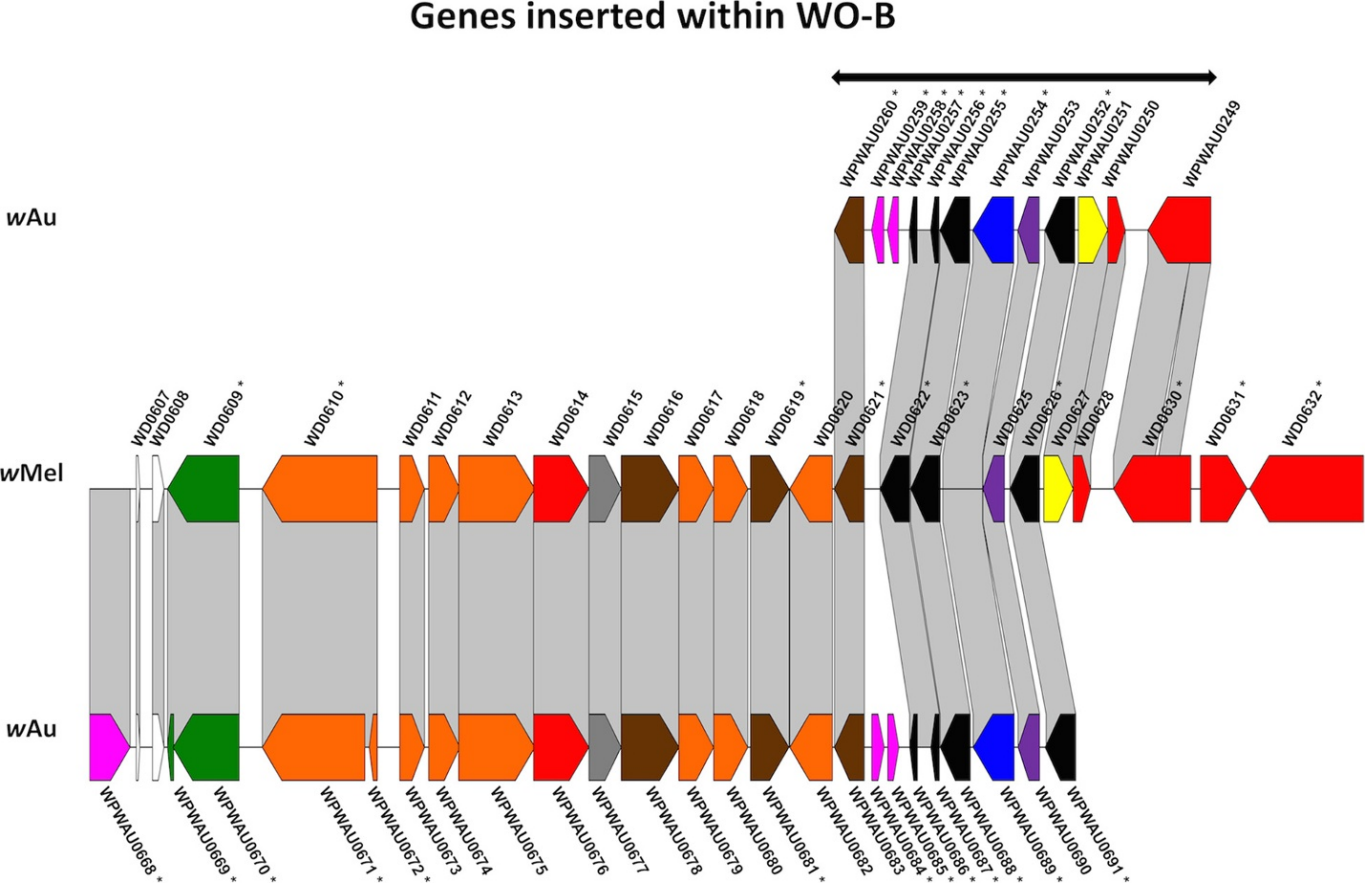
**Comparison of genes inserted within** ***w*** **Mel WO-B in** ***w*** **Au and** ***w*** **Mel.** Alignment of genes inserted within *w*Mel WO-B between *w*Au and *w*Mel, constructed and presented as for Figure 2, with the CDS colour coding: green, phage structural or replication genes; yellow, conserved hypotheticals; red, hypotheticals; blue, ankyrin repeat genes; magenta, transposases or reverse transcriptases; orange, enzyme genes; brown, membrane protein genes; purple, *radC*; black, transcriptional regulator genes; grey, others.

**Figure 5:**
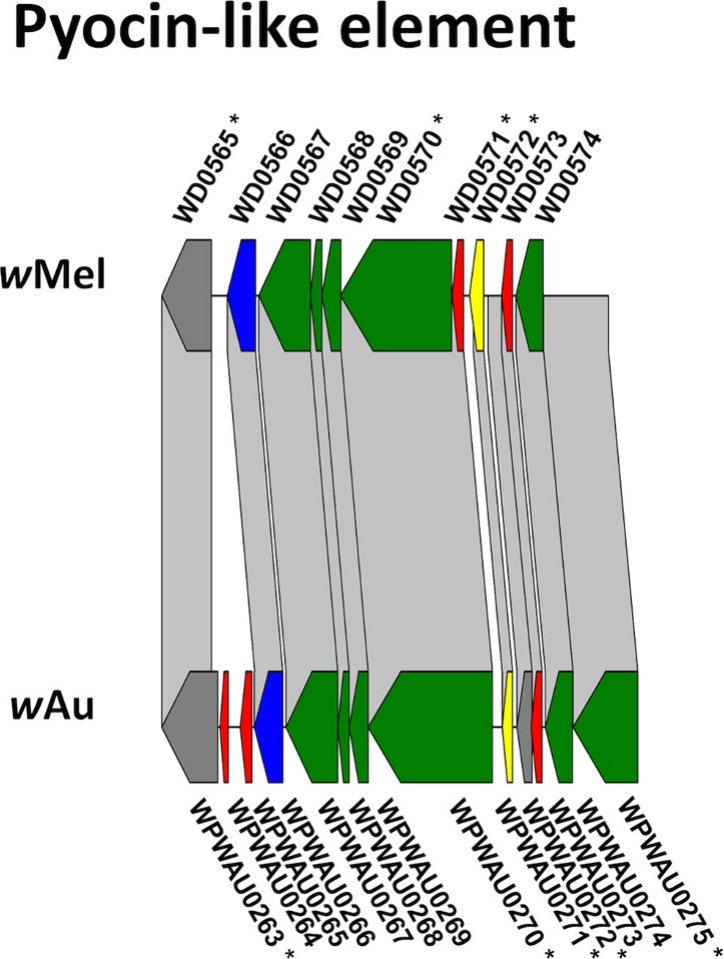
**Comparison of pyocin-like element in** ***w*** **Au and** ***w*** **Mel.** Alignment of the pyocin-like prophage region between *w*Au and *w*Mel, constructed and presented as for Figure 2, with the CDS colour coding: green, phage structural or replication genes; yellow, conserved hypotheticals; red, hypotheticals; blue, ankyrin repeat genes, grey, others.

There are three prophage regions in the *w*Au genome, as for *w*Mel, although the location and structure of these regions differs between the two strains (Figures 2, 3, 4 and 5). The prophage region designated WO-A (Figure 2) in *w*Mel (spanning *WD0259 – WD0294*) is inverted in *w*Au relative to *w*Mel, and is further from the origin of replication (spanning *WPWAU0631 – WPWAU0666*). Several genes in this region differ in one strain relative to the other beyond SNPs (Figure 2). Four genes are disrupted in *w*Au relative to *w*Mel, due to truncation (a shortened gene sequence due to partial deletion or genome rearrangement), frameshift, nonsense mutation, or start codon mutation, two are disrupted in *w*Mel relative to *w*Au, and two contain small in frame indels.

The region designated WO-B (Figure 3) in *w*Mel (spanning *WD0582 – WD0644*) is closer to the origin of replication in *w*Au (spanning *WPWAU0282 – WPWAU0318*). This region contains two segments, one closely related to P2 phage and the other to lambdoid phage [20]. In *w*Au the P2-like segment is inverted relative to its orientation in *w*Mel. In addition, in *w*Mel there are intervening genes between the lambdoid-like block and P2-like block, whereas in *w*Au the two blocks are contiguous. These differences mean that unlike *w*Mel WO-B, the gene order of *w*Au WO-B is highly conserved with that of WO phage in *w*Kue [32], from which WO-A and WO-B were named [20]. As in WO-A, a high proportion of the genes in WO-B are disrupted in one of the two strains. Five genes are disrupted in *w*Au relative to *w*Mel, due to truncation or frameshift; another five are disrupted in *w*Mel relative to *w*Au, due to frameshift, IS element insertion or start codon mutation. Several of the genes between the lambdoid-like and P2-like blocks in *w*Mel, which include three of the transcriptional regulator genes discussed below, also differ significantly between *w*Mel and *w*Au (Figure 4). In addition to a higher than average frequency of SNPs between the two strains, five genes are disrupted in *w*Au relative to *w*Mel, due to truncation, frameshift or nonsense mutation, while two are disrupted in *w*Mel relative to *w*Au. Two genes contain small indels, and two more (*WD0631* and *WD0632*) are absent in *w*Au, discussed further below.

The third prophage region, a small pyocin-like element comprising *WD0565 – WD0574* in *w*Mel (Figure 5), is closer to the origin of replication in *w*Au (comprising *WPWAU0263* – *WPWAU0275*). One gene is disrupted in *w*Au relative to *w*Mel, by a nonsense mutation, and four genes are disrupted in *w*Mel relative to *w*Au, by frameshift, nonsense mutation or truncation. In addition, there is an insertion after the first gene in *w*Au relative to *w*Mel, in which two additional genes are annotated. Overall the level of difference between *w*Mel and *w*Au in these three prophage regions is much higher than elsewhere in the genome.

### Genes potentially inactive in *w*Au

Various other *w*Mel genes were also identified with large differences potentially causing inactivation in *w*Au, or which are absent from *w*Au entirely, as listed in Table 2. It is likely that CI is a complex process involving many genes, with the host genetic background also playing a role [33–35]. Any genes with differences between CI and non-CI inducing strains are possible candidates for involvement in the process, but it seems reasonable to focus more attention on genes that are entirely absent or potentially inactivated. As has previously been reported [19], a region corresponding to genes *WD0506* to *WD0518* is absent in *w*Au; no other indel of comparable size to the *WD0506* to *WD0518* segment was found to be absent in the *w*Au genome. However, of note was the absence in *w*Au of two genes, *WD0631* and *WD0632,* that have recently been identified as CI candidates due to the detection in *Cx. pipiens* spermathecae of a protein corresponding to the *WD0631* ortholog in the *w*Pip strain [36]; the *WD0631* and *WD0632* orthologs are transcribed as an operon [36]. Various other genes are potentially inactivated in *w*Au relative to *w*Mel, by truncation, frameshift, mobile element insertion, nonsense mutation or start codon mutation, as shown in Table 2. Whether and how these differences contribute to CI is worthy of further investigation.

**Table 2:** ***w*** **Mel genes potentially inactive in** ***w*** **Au**

***w***Mel gene	Function	Matching ***w***Au gene(s)	Difference in ***w***Au
*WD0092*	DNA processing chain A	*WPWAU0139*/*WPWAU0140*	Frameshift
*WD0139*	Transcriptional activator, tenA family, putative	*WPWAU0095*	Start codon mutation
*WD0196*	Hypothetical protein	No match	Truncation
*WD0254*	Transcriptional regulator, putative	*WPWAU0256*/*WPWAU0257*/*WPWAU0686*/*WPWAU0687*	Frameshift
*WD0274*	Conserved hypothetical protein	*WPWAU0651*	Start codon mutation
*WD0284*	Conserved hypothetical protein	*WPWAU0640*/*WPWAU0641*	Frameshift
*WD0288*	Prophage LambdaW1, site-specific recombinase, resolvase family	*WPWAU0636*	Nonsense mutation
*WD0294*	Ankyrin repeat domain protein	*WPWAU0631*	Truncation
*WD0295*	Hypothetical protein	*WPWAU0322*/*WPWAU0323*	Frameshift
*WD0382*	Conserved hypothetical protein	*WPWAU0417*/*WPWAU0418*/*WPWAU0419*	Frameshift
*WD0383*	Hypothetical protein	*WPWAU0420*/*WPWAU0421*	Frameshift
*WD0385*	Ankyrin repeat domain protein	*WPWAU0423*/*WPWAU0426*/*WPWAU0427*	Mobile element insertion
*WD0446*	Hypothetical protein	*WPWAU0481*/	Frameshift
		*WPWAU0482*	
*WD0462*	Hypothetical protein	*WPWAU0494*/	Frameshift
		*WPWAU0495*	
*WD0463*	ATPase, AAA family	*WPWAU0496*	Mobile element insertion
*WD0472*	ATPase, AAA family	*WPWAU0507*/	Nonsense mutation
		*WPWAU0508*	
*WD0507*	DNA repair protein RadC, truncation	No match	Absent
*WD0508*	Transcriptional regulator, putative	No match	Absent
*WD0509*	DNA mismatch repair protein MutL-2	No match	Absent
*WD0511*	Conserved hypothetical protein	No match	Absent
*WD0512*	Hypothetical protein	No match	Absent
*WD0513*	Hypothetical protein	No match	Absent
*WD0514*	Ankyrin repeat domain protein	No match	Absent
*WD0548*	Hypothetical protein	*WPWAU0565*	Frameshift
*WD0572*	Conserved hypothetical protein	*WPWAU0271*	Frameshift
*WD0582*	Regulatory protein RepA, putative	*WPWAU0282*/	Frameshift
		*WPWAU0283*/	
		*WPWAU0284*	
*WD0591*	Conserved hypothetical protein	*WPWAU0289*	Frameshift
*WD0594*	Prophage LambdaW4, DNA methylase	*WPWAU0291*/	Frameshift
		*WPWAU0292*	
*WD0609*	Regulatory protein RepA, putative	*WPWAU0669*/	Frameshift
		*WPWAU0670*	
*WD0610*	Helicase, SNF2 family	*WPWAU0671*/	Nonsense mutation
		*WPWAU0672*	
*WD0619*	GlpT/PgpT/UhpT transporter family protein	*WPWAU0681*	Frameshift
*WD0622*	Transcriptional regulator, putative	*WPWAU0256*/	Frameshift
		*WPWAU0257*/	
		*WPWAU0686*/	
		*WPWAU0687*	
*WD0630*	Hypothetical protein	*WPWAU0249*	Truncation
*WD0631*	Hypothetical protein	No match	Absent
*WD0632*	Hypothetical protein	No match	Absent
*WD0636*	Prophage LambdaW5, ankyrin repeat domain protein	*WPWAU0314*/	Frameshift
		*WPWAU0315*	
*WD0638*	Conserved hypothetical protein	*WPWAU0311*/	Frameshift
		*WPWAU0312*	
*WD0682*	Ribosomal protein S10	*WPWAU0607*	Nonsense mutation
*WD0686*	Conserved domain protein	*WPWAU0613*/	Mobile element insertion
		*WPWAU0614*/	
		*WPWAU0618*	
*WD0696*	Hypothetical protein	*WPWAU0698*	Nonsense mutation
*WD0766*	Ankyrin repeat domain protein	*WPWAU0768*	Nonsense mutation
*WD1041*	Surface protein-related protein	*WPWAU1092*/	Frameshift
		*WPWAU1093*/	
		*WPWAU1094*	
*WD1111*	Hypothetical protein	*WPWAU0887*/	Frameshift
		*WPWAU0888*	
*WD1180*	Recombination protein RecR	*WPWAU1182*	Nonsense mutation
*WD1187*	Hypothetical protein	*WPWAU1187*	Frameshift
*WD1320*	Multidrug resistance protein D	*WPWAU1315*	Frameshift

Truncation refers to shortening of the gene sequence due to partial deletion or genome rearrangement. Small hypothetical genes with multiple matches are not included, as these are likely to be remnants of mobile elements. Genes annotated as truncations in *w*Mel are not included, as these are likely to be inactive in *w*Mel.

### Comparison of transcriptional regulator genes

**Figure 6:**
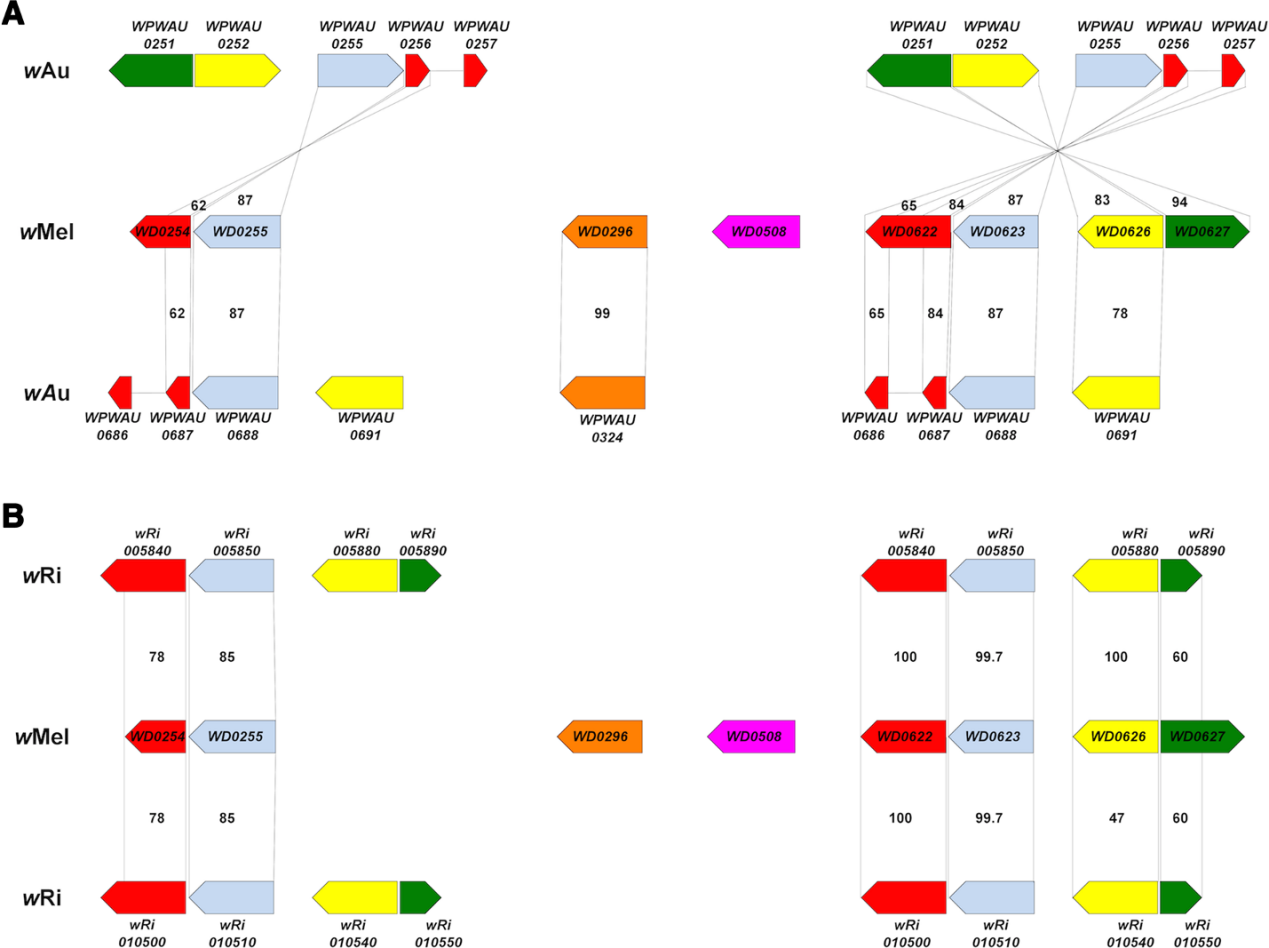
**Homology between transcriptional regulator genes.** An illustration of the *w*Mel transcriptional regulator genes and their homologs in *w*Au **(A)** and *w*Ri **(B)**, identified using ACT and Geneious alignments. Genes depicted in the same colour are thought to be paralogous within an individual strain, and either orthologous or paralogous between strains. Numbers indicate the percentage amino acid similarity between any proteins produced from these genes.

**Figure 7:**
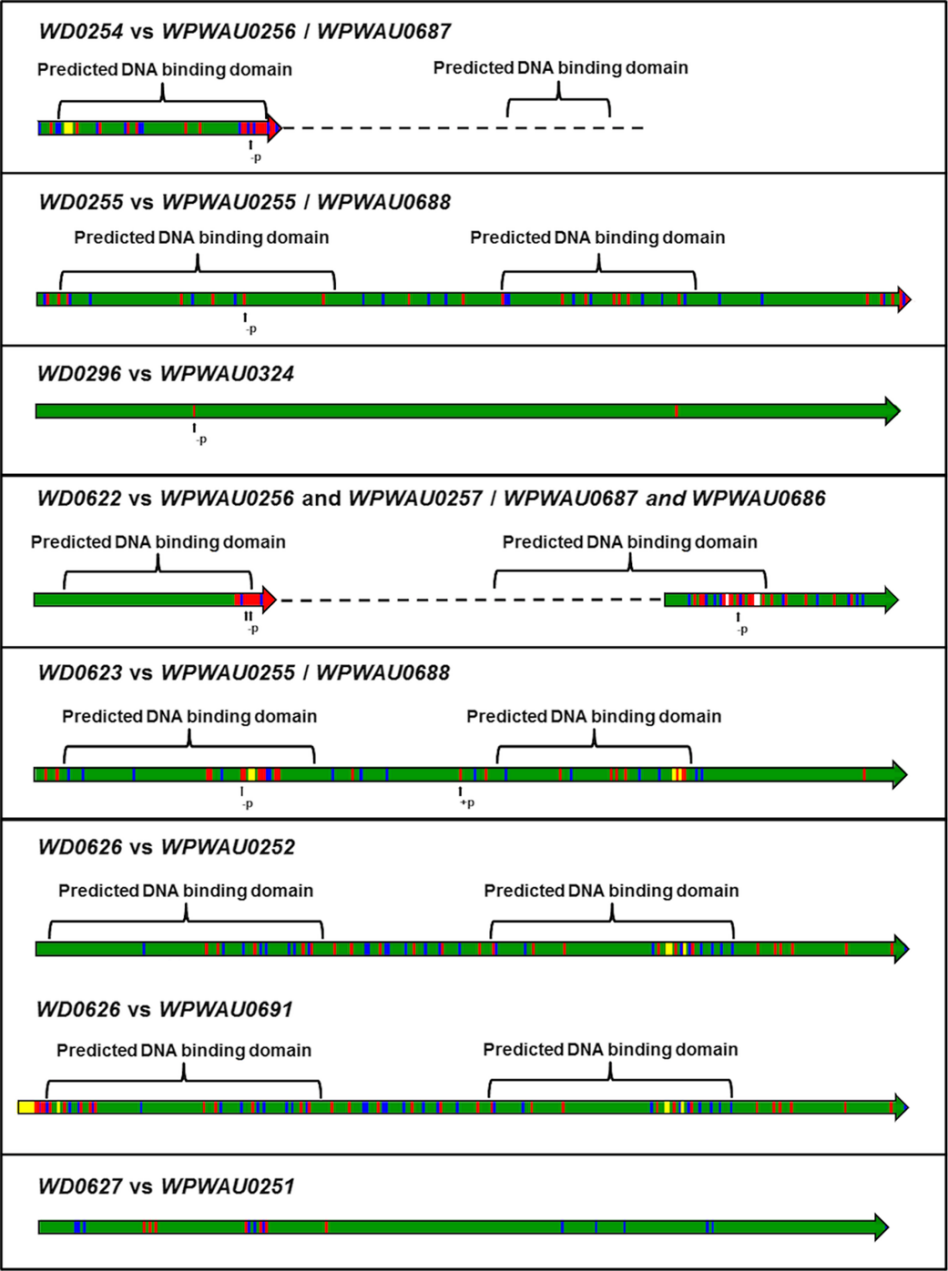
**Differences in proteins from transcriptional regulator genes between** ***w*** **Au and** ***w*** **Mel.** An illustration of the differences between proteins that would be produced from the *w*Mel transcriptional regulator genes and their corresponding sequences in *w*Au. Proteins predicted from the *w*Au sequences are shown. Green indicates identity with the *w*Mel translated sequence. Blue indicates substitution with a similar amino acid. Red indicates substitution with a dissimilar amino acid. Yellow indicates inserted amino acids that are present in the *w*Au protein but not the *w*Mel protein. White indicates deleted amino acids that are present in the *w*Mel protein but not the *w*Au protein. The locations of DNA binding domains predicted from the translated *w*Mel sequences are shown. Substitutions involving proline residues are indicated with arrows. – p: loss of a proline residue; + p: gain of a proline residue.

Following a prior comparative genomic study of sub-strains of *w*Pip *Wolbachia* from *Cx. pipiens* mosquitoes [22], which implicated a transcriptional regular gene designated *wtrM* in CI, a comparison of the family of transcriptional regulator genes between *w*Au and *w*Mel constituted a focus of this study. These transcriptional regulator genes were found to differ in both organisation in the genome and sequence. Figure 6 illustrates the homology between *w*Au and *w*Mel transcriptional regulator genes; also shown is a comparison between *w*Mel and *w*Ri, a CI-inducing strain found in *D. simulans*. Figure 7 illustrates the differences between proteins that would be produced from the *w*Mel transcriptional regulator genes and their corresponding sequences in *w*Au. Of particular note is that the ortholog of *WD0622* is highly disrupted by a frameshift in *w*Au. The two identical sequences corresponding to *WD0622* (*WPWAU0256* and *WPWAU0687*) have a 1 bp insertion in *w*Au relative to *w*Mel, after bp 212, which causes a frameshift that would lead to premature termination of translation after 85 amino acids. Genes corresponding to the last 82 amino acids of *WD0622* (*WPWAU0257* and *WPWAU0686*) are also predicted since a substitution has produced a new start codon, although these may be mis-predictions, as numerous SNPs and deletions have accumulated relative to *WD0622*. None of these genes include the DNA binding domains present in *WD0622*, suggesting that even if they are functional their activity is likely to differ significantly from that of their counterpart in *w*Mel. Furthermore, there are IS5 elements inserted immediately downstream of the *w*Au genes corresponding to *WD0622*, so it may be that the regulation of the genes differs between the two strains. Genes such as this, which are conserved between multiple CI-inducing strains (*WD0622* has two orthologs in the CI-inducing *w*Ri strain) but disrupted in a non-CI strain, are prime candidates for involvement in CI.

In the *w*Mel genome, *WD0254*, *WD0255*, *WD0622*, *WD0623* and *WD0626* are in two regions that appear to be paralogous; *WD0254* is a truncated paralog of *WD0622* (with the truncation appearing to be due to a transposase insertion) and *WD0255* a paralog of *WD0623*. In the *w*Au genome, there also appear to be two genome segments containing paralogous sequences at approximately the same genomic positions as in *w*Mel. However, the segment at a similar position to *WD0254* and *WD0255* is inverted relative to its orientation in *w*Mel and is adjacent to genes that match *WD0628-WD0630* in *w*Mel, suggesting that one or more translocations have occurred, encompassing *WD0622* to *WD0630*; flanking IS elements provide a putative mechanism for its translocation and inversion. Unlike in *w*Mel, in which there are sequence differences between the paralogs in the two paralogous transcriptional regulator regions, in *w*Au *WPWAU0687*, *WPWAU0686* and *WPWAU0688* are identical to *WPWAU0256*, *WPWAU0257* and *WPWAU0255* respectively, while *WPWAU0691* is identical to *WPWAU0252* after the first 84 bp; this suggests that replacement by intragenomic recombination has occurred. *WD0296* appears to have an ortholog in *w*Au, *WPWAU0324*, in a similar genomic position, while there is no ortholog of *WD0508* present in *w*Au, consistent with a previous study that found that the region spanning *WD0506* to *WD0518* in *w*Mel is absent from *w*Au [19]. This gene is also absent in several other CI-inducing *Wolbachia* strains [19].

Analysis of the other transcriptional regulator gene sequences indicates that protein products from these genes would be different between *w*Au and *w*Mel (Figure 7), particularly *WD0623* and *WD0626*. The two identical *w*Au genes corresponding to *WD0623* (*WPWAU0255* and *WPWAU0688*) both have three small insertions in the putative DNA binding domains that result in an extra four amino acids and one amino acid substitution; there are 37 further amino acid substitutions, 23 of which are located in the putative DNA binding domains. There are substitutions involving proline residues; due to the unique conformational rigidity of the proline side chain, this could have a large impact on the secondary structure of any protein produced, and thus probably also its function. There are also SNPs and a 6 bp insertion in 5′ upstream regions where promoter elements have been shown to occur in prokaryotes, centred at −45 and −52 [37]. In the *w*Au homolog of *WD0626*, *WPWAU0252*, two small insertions in the putative DNA binding domains would result in an extra three amino acids and one amino acid substitution. In addition there are 48 amino acid substitutions, 27 of which are located in the putative DNA binding domains. There are also SNPs and a 16 bp deletion in the 5′ upstream region spanning the −45 and −52 positions. Another factor that may affect expression is their different genomic location; in *w*Mel, *WD0622*, *WD0623* and *WD0626* are located within a prophage region, whereas in *w*Au this is not the case. The precise impact of all these differences described on protein function is hard to predict, but given in particular the changes in DNA binding domains it seems highly likely that their activity will be affected.

**Figure 8:**
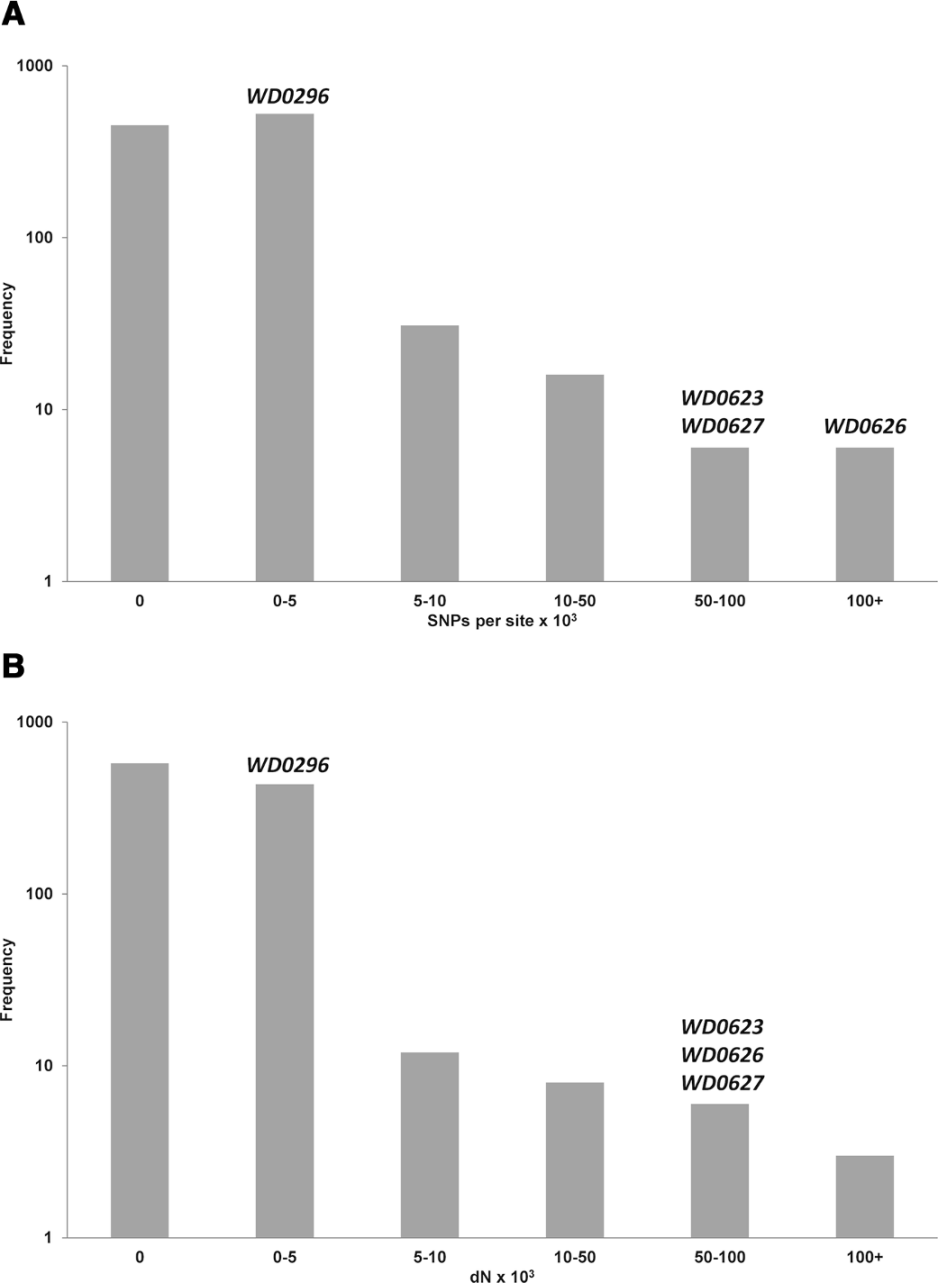
**Frequency distribution of SNP density and dN.** Graph showing the number of protein-coding genes in the *w*Mel genome within each SNP density **(A)** and dN **(B)** category. The frequency axis is drawn on a log scale. The bins containing the transcriptional regulator genes included in the analysis are indicated. Pseudogenes, genes that are potentially inactivated in *w*Au, IS elements and other genes with multiple ambiguous matches are excluded. The transcriptional regulator gene *WD0255* is excluded as its closest *w*Au sequence contains a frameshift and is more similar to *WD0623*. dN – number of non-synonymous SNPs per potential site.

**Figure 9:**
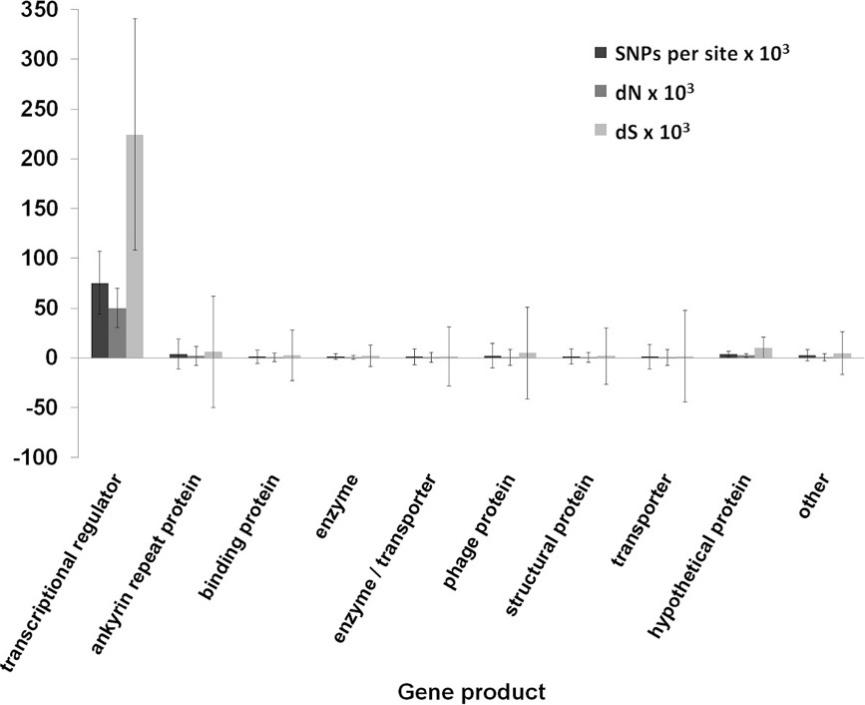
**Mean SNP density, dN and dS by gene category.** Graph showing the mean SNP density, dN, and dS for different categories of protein-coding gene in the *w*Mel genome. Error bars represent the standard error of the mean. Pseudogenes, genes that are potentially inactivated in *w*Au, IS elements and other genes with multiple ambiguous matches are excluded. The transcriptional regulator gene *WD0255* is excluded as its closest *w*Au sequence contains a frameshift and is more similar to *WD0623*. dN – number of non-synonymous SNPs per potential site. dS – number of synonymous SNPs per potential site.

The total SNP density and the density of non-synonymous SNPs (dN) for *WD0623* and *WD0626* relative to their *w*Au orthologs are at the extreme end of the distributions of these measures over the genome (Figure 8). Comparing these measures between gene categories shows that the transcriptional regulator genes as a whole have a much higher density of total SNPs and non-synonymous SNPs than all other categories, as well as a higher density of synonymous SNPs (dS) (Figure 9). A Kruskal-Wallis test shows a statistically significant difference between groups for total SNP density, dN and dS (P <0.01 in all cases), and post-hoc pairwise Wilcoxon tests with Benjamini-Hochberg correction show a statistically significant difference in total SNP density and dN between the transcriptional regulator genes and all other categories except genes for ankyrin repeat proteins (P <0.05 in all cases). dS was not significantly different between transcriptional regulator genes and other groups, except the structural protein and hypothetical protein groups. These findings suggest that the transcriptional regulator genes may be under positive selection.

To confirm whether any of these transcriptional regulator genes are involved in CI, functional studies are required, although these are currently difficult to undertake in the absence of an effective *Wolbachia* transformation methodology. Expression vectors have been used for transfection and shown to change transcription levels of a host cell cycle regulator, but their tissue distribution is uneven, limiting their use for examining whether a CI-like phenotype can be induced [22]. The best approach may be to transform hosts with target *Wolbachia* genes and assess whether a CI phenotype can be induced, as has been performed for ankyrin repeat-encoding genes previously [38], although if multiple interacting *Wolbachia* genes are required to produce the CI phenotype the use of individual genes in this way may not clearly reveal their role. As more *Wolbachia* genomes are sequenced, further comparison of these genes between different strains will also be useful.

## Conclusions

In this study, a methodology for conveniently extracting *Wolbachia* gDNA for genome sequencing using an infected cell line has been successfully employed, and the PacBio RS II sequencing platform has proved a very useful tool for achieving a complete bacterial assembly, particularly when combined with Illumina sequencing. Using this approach, a single contig assembly has been generated for the genome of the *w*Au strain, which does not induce CI. Comparison of this genome to that of *w*Mel, which does induce CI, revealed significant structural differences in the prophage regions and loss or potential inactivation of a number of genes. Transcriptional regulator genes in particular displayed considerable differences between *w*Au and *w*Mel, both in terms of genomic location and sequence; of these the *w*Mel gene *WD0622* may be the most promising to examine as a CI candidate. Given that a transcriptional regulator gene has previously been implicated in CI, these represent important targets for further functional studies on the mechanism of CI. The *w*Mel genome region containing the transcriptional regulator genes *WD0622*, *WD0633*, *WD0626* and *WD0627* is also of particular interest with respect to CI given the proximity of *WD0631-2*, absent in *w*Au, because a *WD0631* ortholog protein was recently identified in mosquito spermathecae. The current study thus contributes to the important basic aim of gaining a better understanding of the molecular basis of CI.

## Methods

### *Drosophila*rearing

*D. simulans* flies infected with *w*Au from Coffs Harbour, Australia, were reared using standard techniques. Flies were maintained at 25°C with a 12:12 hour light: dark cycle, in plastic bottles containing 25 ml food, and transferred to fresh containers when necessary.

### Cell culture

*Aedes albopictus* Aα23 cells infected with *w*Au were maintained using standard cell culture techniques. Cells were maintained in an incubator at 28**°**C, in flasks containing 10 ml Schneider’s medium with 10% fetal bovine serum, penicillin and streptomycin, and passaged when required.

### Genomic DNA extraction

gDNA for genome sequencing was extracted using two different methods. To extract gDNA from whole flies, a slightly modified version of the protocol used by Iturbe-Ormaetxe *et al*. [27] was used. Approximately 10–25 ml flies were collected, sterilised in 50% bleach for 3 minutes, rinsed in filter-sterilised dH_2_O, then further sterilised in 70% ethanol and rinsed again in filter-sterilised dH_2_O. The flies were then homogenised in cold SPG buffer (3.8 mM KH_2_PO_4_, 4.9 mM L-glutamate, 7.2 mM K_2_HPO_4_, and 218 mM sucrose) using a Polytron homogeniser (Kinematica, Switzerland). After homogenisation the sample was centrifuged at 3,200 g for 15 minutes. The supernatant was collected and the centrifugation repeated. The supernatant from the second centrifugation was sequentially filtered through 5 μm, 2.7 μm and 1.2 μm syringe filters. The filtrate was centrifuged at 18,000 g for 20 minutes to pellet *Wolbachia*, which were resuspended in cold SPG buffer. The suspension was then incubated with 600 ng of DNase I (Roche, UK) at 37**°**C for 30 minutes and subsequently with 5 μl of RNase A (Fermentas, UK) at 37**°**C for 15 minutes to remove host DNA and RNA contamination. Cells were then lysed by incubation with 200 μg of proteinase K (Sigma-Aldrich, UK) at 56**°**C. gDNA was purified using two phenol/chloroform/isoamyl alcohol extractions and one chloroform/isoamyl alcohol extraction.

To extract gDNA from cells, *Wolbachia* were first purified from the cells. Cells were dislodged from flasks by pipetting and scraping, and lysed by vortexing with borosilicate beads. The lysate was centrifuged at 2,500 g for 10 minutes at 4**°**C, then filtered sequentially through 5 μm and 0.2 μm filters. Sucrose gradient centrifugation was performed at 18.500 g for 10–20 minutes at 4**°**C to pellet the *Wolbachia*. gDNA was purified using the method described by Livak [39]. To extract gDNA for PCR, the Livak method was used on adult *w*Au-infected flies.

### Genomic DNA purity assessment

Extracted *w*Au gDNA was analysed for contamination with host gDNA using qPCR. Reactions were performed on five serial dilutions of the extracts, using primers specific for a *w*Au gene (*wsp*), a host nuclear gene (*RpL32* for *D. simulans* and *hth* for *Ae. albopictus*) and a host mitochondrial sequence (mitochondrial rRNA). The average relative C(t) values for each primer pair, corrected for differences in primer efficiencies, were calculated. Taking into account the different sizes of the genomes, these values were compared to give a ratio of the amount of *w*Au gDNA to host gDNA, and the figures in this ratio were converted into percentages.

### Genome sequencing and assembly

*w*Au gDNA extracted from whole files was sequenced using the Illumina HiSeq 2000 platform. A ~200-300 bp paired end library was constructed following the methods described by Quail *et al*. [40, 41], using Kapa HiFi polymerase for PCR to reduce GC bias [42]. The library was given a unique index and sequenced as part of a lane with other samples. 357 Mb of data was generated from 3,565,172 reads of 100 bp. An assembly was generated from these reads with Velvet [43], using the *w*Mel genome as a reference. The final assembly comprised 77 contigs, with a total length of 1,222,634 bp, an N50 of 29.5 kb and a mean coverage of 283×.

*w*Au gDNA extracted from cells was sequenced using the PacBio RS II platform. A ~10 kbp library was constructed following standard protocols using a PacBio DNA Template Prep Kit. Three SMRT® cells from this library were sequenced, with a movie length of 2 hours. With filters set to exclude reads of quality <0.8, polymerase read length <500 bp and sub-read length <500 bp, 139 Mb of data was generated from 75,456 sub-reads (from 39,514 polymerase reads), with a mean sub-read length of 1,847 bp. A *de novo* assembly was generated from these sub-reads using the Hierarchical Genome Assembly Process (HGAP) version 1.0 [44], with the genome size parameter set to 1.2 Mb. This resulted in an assembly comprising a single contig of 1,273,534 bp, with a mean coverage of 62×.

Errors in the assembly were corrected using the data from the Illumina sequencing. The Illumina reads from the second sample were mapped to the assembly using SMALT [45], then the assembly sequence was modified based on the mapped reads using Iterative Correction of Reference Nucleotides (iCORN) [46] with four iterations, resulting in the correction of 1 SNP and 88 indels.

### Genome annotation

The assembly was annotated using the Automated Annotation Pipeline at the Wellcome Trust Sanger Institute, with the software Prokka [47]. Infernal [48] was used to identify RNA structures, followed by ARAGORN [49], Rnammer [50] and Prodigal [51] to identify transfer RNAs (tRNAs) and transfer messenger RNAs (tmRNAs), ribosomal RNAs (rRNAs) and proteins, respectively. The predicted genes were compared against *Wolbachia* sequences from RefSeq [52], using CD-hit [53] to create a non-redundant protein database, then against UniProtKB/SwissProt [54]. Some annotations were edited manually.

### Comparative analysis

The *w*Mel and *w*Au genomes were compared using Artemis Comparison Tool (ACT) [55]. Sequences of individual genes of interest were extracted and alignments and translations generated using Geneious 7.0.5, created by Biomatters [56]. Predicted protein domains were identified using InterPro [57]. SNP analysis was performed with the aid of Synonymous Non-synonymous Analysis Program (SNAP) v1.1.1 [58, 59]. The Gene Ontology (GO) project [60] was used to aid categorisation of genes. The dot plot comparing *w*Au and *w*Mel genomes was generated using Dotter [61] with default parameters.

### Sequence confirmation

The sequences of regions of interest were confirmed using PCR. Primers were designed to flank the regions of interest in the *w*Au genome. Amplification was performed using standard PCR conditions. PCR products were run on an agarose gel to check their size, then purified using a Qiagen PCR purification kit and sequenced using GATC Biotech sequencing.

### Availability of supporting data

The *w*Au genome sequence has been submitted to the EMBL/GenBank/DDBJ database with the accession number LK055284. The raw Illumina sequence reads have been submitted to the European Nucleotide Archive with the accession number ERS151014.

## Abbreviations

ACT: Artemis comparison tool
bp: Base pair
CDS: Coding sequence
CI: Cytoplasmic incompatibility
*Cx*: Culex
*D*: Drosophila
gDNA: genomic DNA
HGAP: Hierarchical Genome Assembly Process
iCORN: Iterative Correction of Reference Nucleotides
IS: Insertion sequence
qPCR: Quantitative polymerase chain reaction
PCR: Polymerase chain reaction
rRNA: Ribosomal RNA
SEM: Standard error of the mean
SNAP: Synonymous Non-synonymous Analysis Program
SNP: Single nucleotide polymorphism
tRNA: Transfer RNA
tmRNA: Transfer messenger RNA.
